# Supplementary material for: Nitrite Concentration in the Striated Muscles Is Reversely Related to Myoglobin and Mitochondrial Proteins Content in Rats
Source: Int J Mol Sci. 2022 Feb 28;23(5):2686. doi: 10.3390/ijms23052686 (PMC8910716; doi:10.3390/ijms23052686)
Supplement: Supplementary file 1 [file ijms-23-02686-s001.zip › ijms-1605636-supplementary.pdf]

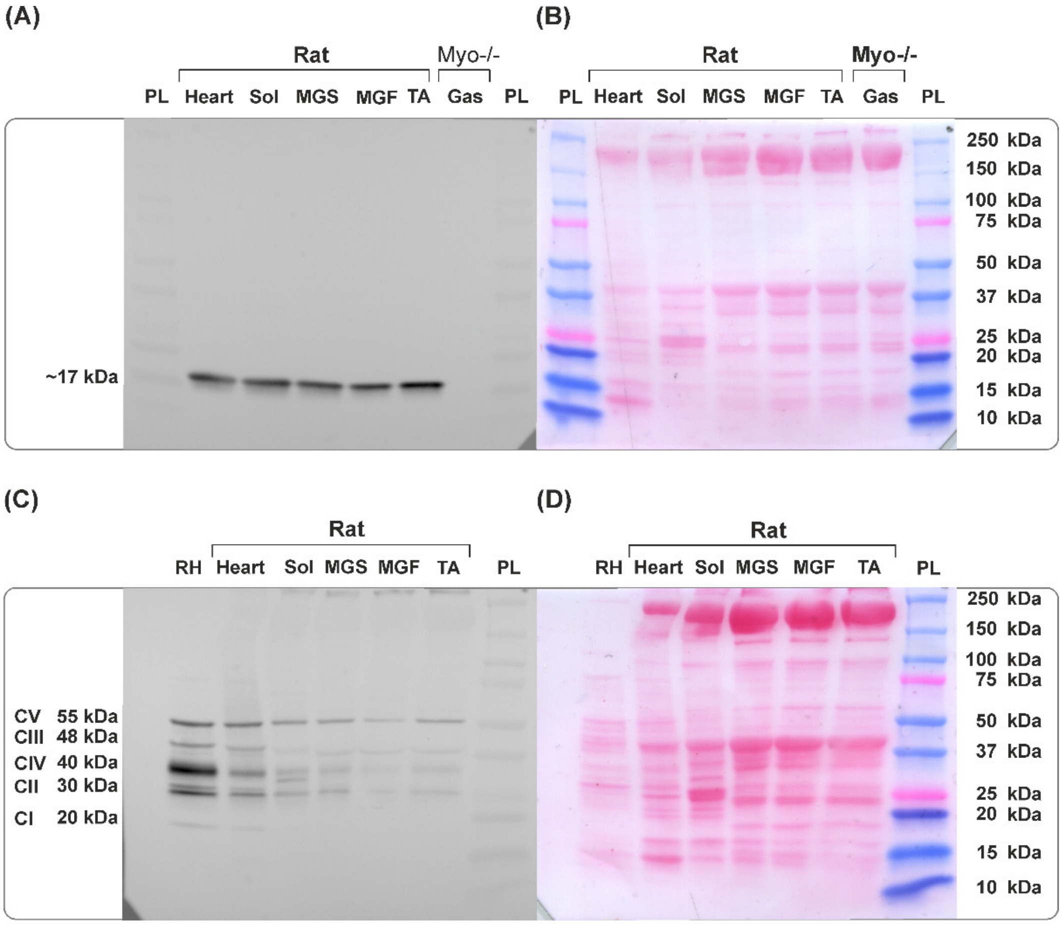

Figure S1. Myoglobin and electron transport chain protein subunits (ETC proteins) in the muscle samples. Representative immunoblot demonstrating detection of myoglobin protein expression with anti-myoglobin antibody (Cat#ab77232, Abcam, Cambridge, UK) in protein extracts derived from the heart, soleus (Sol), slow part of medial gastrocnemius (MGS), fast part of medial gastrocnemius (MGF) and tibialis anterior (TA) of rats and from the gastrocnemius (GA) of myoglobin knockout mice (Myo-/-) (panel A). Ponceau-S staining of the same membrane demonstrating total protein (panel B). Representative immunoblot demonstrating detection of the subunits of mitochondrial complexes expression with anti-ETC protein subunit antibodies (Cat#ab110413, Abcam, Cambridge, UK) in protein extracts derived from the heart, Sol, MGS, MGF and TA of the rat (panel C). Lane 1 contains rat heart mitochondrial lysate (RH) as the positive control (ab110341). The protein ladder (PL) is a visible Precision Plus Protein Dual Colour Standards (Biorad, Cat#1610374). Ponceau-S staining of the same membrane demonstrating total protein loaded (panel D).

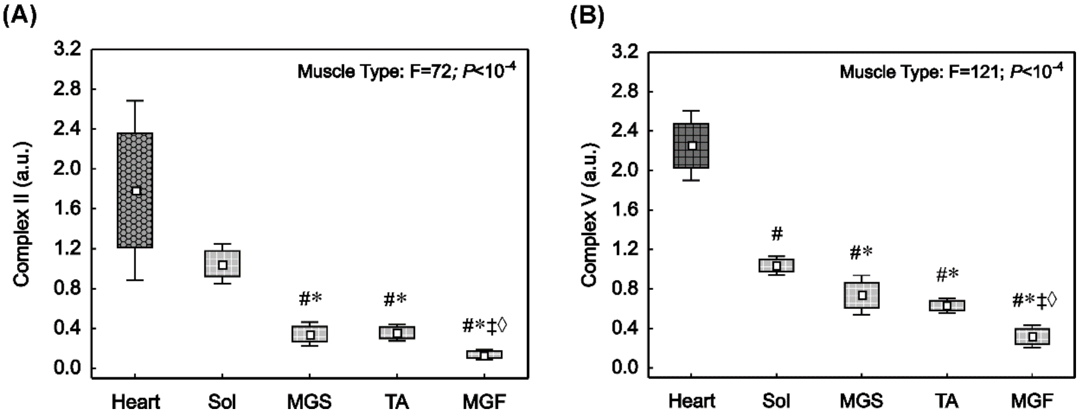

Figure S2. Mitochondrial complex II and complex V protein contents in rat striated muscles with varied muscle fibre type composition. Complex II protein content (Cat#ab14714, (**A**)) and complex V protein content (Cat#ab14748, (**B**)) in the heart (dark square) and in the locomotory muscles (bright squares) i.e., soleus (Sol), slow part of medial gastrocnemius (MGS), tibialis anterior (TA) and fast part of medial gastrocnemius (MGF). Boxes and whiskers represent, correspondingly, the 95% confidence intervals for means and the standard deviations of 12 animals for each studied muscle tissue (i.e., heart, Sol, MGS, TA and MGF). The impact of muscle type on the analysed variables is presented. Results of Welch ANOVA and post-hoc Games-Howell analysis are presented. # denotes significant difference to the heart; * denotes significant difference to Sol; ‡ denotes significant difference to MGS; ◊ denotes significant difference to TA.

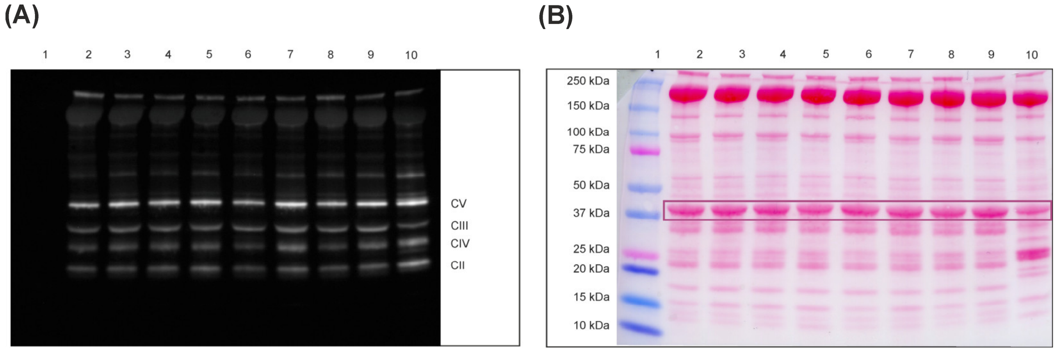

Figure S3. Western blot detection of electron transport chain protein subunits (ETC proteins) in rat muscle samples. Representative immunoblot membrane demonstrating the expression of individual subunits of four visible mitochondrial complexes (CII–CV) in the slow part of medial gastrocnemius (MGS) labelled with anti-ETC protein subunit antibodies (Cat#ab110413, Abcam, Cambridge, UK) (**A**). Ponceau-S staining of the same membrane demonstrating total protein loaded and bands outlined in violet used for normalisation of the signal shown in panel A (**B**). Lane 1 contains the protein ladder (PL) (Precision Plus Protein Dual Colour Standards (Biorad, Cat#1610374). Lines 2–9 were equally loaded with protein extracts (20 µg) derived from the slow part of rat medial gastrocnemius (MGS), whereas line 10 contains the internal standard (20 µg).
